# Creation of a sustainable longitudinal women in Leadership Development (WILD) curriculum focused on graduate medical education trainees

**DOI:** 10.1186/s12909-024-05369-3

**Published:** 2024-04-05

**Authors:** Colleen A. McGourty, Francine Castillo, Grace Donzelli, Bridget P. Keenan, Margaret Gilbreth, Lekshmi Santhosh

**Affiliations:** University of California, San Francisco, 505 Parnassus Ave., 94143 San Francisco, CA USA

**Keywords:** Women, Leadership, Women in leadership, Graduate medical education, Mixed-methods, Gender equity

## Abstract

**Background:**

Although women comprise the majority of medical students, gender disparities emerge early and remain at the highest levels of academia. Most leadership courses focus on faculty or students rather than women graduate medical education (GME) trainees.

**Aim:**

To promote the leadership development of women GME trainees through empowerment, community building, networking and mentorship, and concrete leadership skills development.

**Setting:**

University of California, San Francisco.

**Participants:**

359 women residents and fellows from 41 specialties.

**Program description:**

A longitudinal curriculum of monthly workshops designed to support leadership development for women trainees. Sessions and learning objectives were designed via needs assessments and literature review.

**Program evaluation:**

A mixed-methods evaluation was performed for 3 years of WILD programming. Quantitative surveys assessed participant satisfaction and fulfillment of learning objectives. Structured interview questions were asked in focus groups and analyzed qualitatively.

**Discussion:**

23% of invited participants attended at least one session from 2018 to 2021, despite challenging trainee schedules. Surveys demonstrated acceptability and satisfaction of all sessions, and learning objectives were met at 100% of matched sessions. Focus groups highlighted positive impact in domains of community-building, leadership skills, mentorship, and empowerment. This program has demonstrated WILD’s longitudinal sustainability and impact for women trainees.

**Supplementary Information:**

The online version contains supplementary material available at 10.1186/s12909-024-05369-3.

## Introduction

Despite rising numbers of women medical students and residents, gender disparities persist throughout multiple levels of leadership [1]. Women are less likely to advance to Full Professor, despite adjusting for research productivity [2]. Disparities for women in medicine emerge as early as residency and fellowship, and are particularly notable within procedural specialties [3–5]. Progress is not occurring nearly quickly enough, as it may take decades to achieve gender parity in some surgical specialties [3]. The COVID-19 pandemic has exacerbated inequities for women in medicine [6, 7].

Leadership development programs for academic medicine faculty have helped reduce gender disparities. The Executive Leadership in Academic Medicine program for mid-career faculty focuses on leadership skill development, mentoring, and networking, and has been linked to significant improvements in career progression and perceived leadership capabilities [8]. The Leadership Program for Women Faculty for early-career faculty at Johns Hopkins University, a longitudinal series of seminars and small groups, improved self-perceived leadership skills and negotiation behaviors [9]. However, most programs target women faculty: few programs exist for medical trainees and they are narrower in scope [10, 11] Career development support for residents and fellows is one important approach to stem the well-established “leaky pipeline” from medical school to senior faculty.

We developed an innovative leadership development program for women graduate medical education (GME) trainees inclusive of residents and fellows across all specialties at the University of California San Francisco (UCSF). We describe the creation and sustainability of this GME-wide leadership program that has sustained over the last 5 years in this Innovation Report.

## Setting and participants

GME trainees including all women or non-binary-identifying residents and fellows at UCSF from 2018 to 2021 were invited to participate. The curriculum was delivered in-person at the UCSF campus initially and was transitioned to virtual during the early COVID-19 pandemic.

## Program description

We aimed to design a rigorously developed longitudinal program for women residents and fellows to build community and develop concrete leadership skills. Women in Leadership Development program (WILD) is a yearlong longitudinal program for women residents and fellows across departments that serves as an early intervention to support equitable career advancement for women in medicine. It equips trainees with skills necessary to advance in the leadership pipeline by centering the experiences of women trainees, increasing early career mentorship and professional networks, and supporting the development of concrete leadership skills.

The curriculum was developed and iteratively refined from 2017 to 2018 using the Kern model of curriculum development [12]. We conducted a literature review to define critical competencies for leadership development and performed an internal needs assessment to better understand unique needs of GME trainees. Our internal needs assessment noted that many specialties had discussions on “How to Lead a Team” but not formal leadership training. Our literature review and our internal needs assessment processes identified four guiding pillars in prospectively designing a program to reduce leadership disparities for women: community building, empowerment, concrete leadership skills, and mentorship and networking. Within these pillars, 15 key topics were defined and used as a framework to design each WILD session (Table 1). Resources used in the development of individual session curricula are described in Supplemental Table 1.

The structure and content of the WILD program were rooted in professional identity formation and feminist theory. Though no one was excluded from participating, effort was made to promote WILD as a space celebratory of cis- and transgender women as well as non-binary individuals. Recognizing that feminist programming often centers cisgender white women, integration of the principles of intersectional feminism underscored curriculum development. For example, sessions on navigating microaggressions and promoting allyship were included and facilitated by an external professional consulting firm focused on diversity, equity, and inclusion.

WILD is modeled on successful examples of leadership development programs and uses a combination of large-group lectures, small-group workshops, and networking sessions. To accommodate inflexible trainee schedules, a modular format was chosen in which the curriculum was divided into fifteen one-hour sessions, each with different dedicated topic which were held once every one to two months. Each was designed to function as part of a longitudinal curriculum but also a stand-alone event so that all trainees could participate even if they could only attend one session. This format also allowed for nimble adaptation of the curriculum in response to changing needs and current events such as the COVID-19 pandemic, where specific sessions were added to address topical concerns, such as voting rights and the impact of the pandemic on women in medicine, whereas the core sessions were repeated annually.

Based on trainee preferences, WILD events were held on weekday evenings. Free time to socialize and refreshments were provided prior to each session to promote community building and networking. The program was transitioned to a virtual format using the Zoom videoconferencing platform during the COVID-19 pandemic. Events were publicized via email list-serve to all UCSF GME trainees including residents and fellows. Sessions were primarily facilitated by UCSF faculty with complementary input from external topic experts.

## Program evaluation

Over the first three years of the program, we administered surveys assessing (1) participant demographics, (2) session acceptability, and (3) preliminary session effectiveness. To assess acceptability, we asked participants to evaluate the organization, usefulness, and likelihood of recommending the program to a friend on a 5-point Likert scale.

To assess session effectiveness, we administered surveys assessing trainee confidence in a range of critical leadership competencies before the start of each academic year’s program and after each individual evening session, in addition to end-of-year surveys. Surveys were pilot tested to ensure clarity of questions. Several of these leadership competencies were directly paired with individual WILD sessions (e.g. negotiating for a promotion/raise, navigating microaggressions, understanding family leave rights). Some sessions which were added mid-program in response to evolving trainee needs (e.g. “How the Pandemic is Impacting Women) were not included in baseline surveys and thus effectiveness was not measured.

We assessed barriers to participation in the WILD program through a survey item asking participants to select the most significant barrier from a multiple-choice list, and through focus groups with participants at the end of the program. Two focus groups of 8 participants at different levels of training were conducted after each academic year’s program conclusion in 2019 and 2020. A member of the study team facilitated each group (CM, FC), using a semi-structured interview guide based on a literature review of gender equity program evaluations both within and outside of medicine (Supplemental Table 2). Open-ended questions were used to initiate discussion on the perceived benefits and limitations of the program to individual’s leadership development. After these interviews, we achieved thematic saturation. Interviews were recorded and transcribed using a professional transcription service, Rev.com.

### Conceptual framework

Given the complexity of women in leadership programs in the literature, we selected the conceptual frameworks of professional identity formation and feminist theory. We used these frameworks to develop our interview questions and code book.

### Quantitative data analysis

A combined analysis of effectiveness items was performed in which mean confidence in leadership competencies were calculated for each timepoint and compared using a two-tailed student’s t-test. Statistical significance was pre-defined as *P* < 0.05. A sub-analysis was also performed restricting analysis to participants who attended multiple sessions.

### Qualitative data analysis

De-identified transcribed data were analyzed using a summative content analysis approach. A topic codebook was developed guided by program learning objectives and codes were applied to study transcripts (Version 9.0.17; Dedoose; Los Angeles, CA). Two members of the study team (LS, FC) independently reviewed the coded transcripts to define the final themes and sub-themes. Differences in codes were reviewed and adjudicated for reconciliation.

## Outcomes

23% (359/1560) of all eligible trainees at UCSF elected to participate in at least one session (Supplemental Table 3). Participants represented 41 specialty and subspecialty departments, with internal medicine and its subspecialties the most represented (53%, *n* = 153/291). The racial/ethnic makeup of WILD participants approximately matched that of UCSF GME trainees overall. For participants who completed demographic surveys, residents (39%, *n* = 112/291) and fellows (43%, *n* = 124/291) were similarly represented. The quantitative survey response rate was 81% (291/359) on baseline and post-session surveys, and 19% (69/359) on end-of-program surveys. Most participants (80%, *n* = 233/291) attended only one event, while a minority (20%, *n* = 61/291) attended two or more (Supplemental Table 4). The average number of participants at each event was 25 and ranged from 6 to 79 (Supplemental Table 5).

### WILD session acceptability

Trainees expressed strong satisfaction with WILD sessions. Combining survey data over all sessions, 98% of participants agreed or strongly agreed that WILD sessions were organized and the structures were clear. 97% agreed or strongly agreed that WILD sessions were useful, and 98% agreed or strongly agreed that they would recommend WILD sessions to a friend. “Microaggressions”, “Allyship”, “Building your Personal Board of Directors”, and “Fertility” were the most highly rated sessions.

### Preliminary WILD session effectiveness

At baseline, participants expressed varying degrees of confidence in critical leadership competencies (Table 2). Participants reported most confidence in competencies which are core parts of medical training such as working in teams, providing feedback, and presentation skills. They reported least confidence in competencies related to personal advocacy and gender-specific challenges including employment negotiation, family leave rights, dealing with microaggressions, and garnering political influence. For each of these low-confidence leadership skills, attending the associated WILD session was associated with a significant increase in reported confidence levels by trainees (Table 2).

### Barriers to participation

At the end of the program, participants indicated that the most significant barriers to participation were “having enough time” (29%, 18/64), “inconvenient location” (24% 15/64), and “conflicting clinical responsibilities” (44%, 27/64). Most preferred evening meeting times. When asked how holding WILD sessions exclusively on Zoom would affect their participation, 82% (56/68) of respondents indicated that they would be more likely, or much more likely to attend virtual sessions. In practice, however, WILD attendance at virtual sessions was not significantly different than in-person sessions.

### Focus group themes

The focus group interviewees represented all stages of training, from residency to fellowship, during their participation in the program. Focus group discussions generated numerous themes regarding how participation in WILD-GME facilitated trainees’ leadership development. Participants noted recurrent themes such as how WILD facilitated a safe space to form community among women trainees, how they felt empowered by the curriculum and appreciated the structured leadership training, and how they appreciated the mentorship and networking aspects of the curriculum. Representative quotes are included in Supplementary Table 6.

## Discussion

Nearly one quarter of eligible trainees at UCSF participated in the WILD program at least once over a three-year period. To our knowledge this makes WILD the largest and longest-running program designed to specifically support female medical trainees. Previously published programs for women GME trainees are limited to a specific specialty or level of training. Moreover, a recent systematic review highlighted that there was a significant absence in publication of rigorously designed and evaluated leadership training programs in GME [13]. By contrast, WILD was prospectively rigorously designed and was attended by participants in nearly all clinical departments at UCSF and included over 40% subspecialty fellows - a historically difficult population to target with opt-in interventions. The strong desire amongst trainees for leadership development programming is underscored by the fact that so many women choose to attend these events despite the constraints of rigorous training schedules.

Our results suggest that the WILD curriculum appropriately targets the leadership skills which trainees had self-identified as areas for growth. Participants expressed the most confidence in skills which are already core competencies of medical training (e.g. working in teams, providing feedback), and the least confidence in personal advocacy skills which are not directly addressed in standard GME training (e.g. career negotiations, responding to microaggressions, navigating their rights related to childbearing and family leave). These skills are often interpreted societally through a gender-specific lens and may also drive career inequities for women in medicine and other fields. WILD provides specific content designed to support trainees in the development of these skills. These sessions were successful at increasing self-reported trainee confidence in these leadership domains assessed immediately following their associated session. Based on these findings, we conclude that further iterations of the WILD curriculum or similar programs at other institutions should strongly focus on content that supports the development of personal advocacy skills for women GME trainees.

## Limitations

Although participation in WILD was diverse across subspecialties and levels of training, one limitation is that most only attended one or two sessions and thus were only exposed to a fraction of the curriculum. Thus, our original design as a year-long longitudinal leadership development program was difficult to execute as an elective extracurricular offering to be voluntarily attended by trainees after rigorous 80-hour-work-weeks. In our post-program evaluations, participants indicated that time and travel were major barriers to participation, and that a fully virtual format may increase their participation. Survey results could be influenced by response bias if only highly motivated participants chose to complete surveys. Moreover, the participants in our program self-identified as women; further analysis could explore if non-binary individuals did not feel included by the titling of the event as WILD. The intermittent participation limits the ability to draw meaningful conclusions about the impact of the program on skill development, and lack of a longitudinal component of evaluation limits identification of outcomes such as capturing leadership trajectories.

Despite these limitations, we are struck that nearly a quarter of eligible trainees at UCSF participated in this program volitionally outside of work hours and that the curriculum is still sustainable over 5 years after original implementation, a feat for GME programming. One ideal but logistically challenging solution is to conduct the entire program during protected time in a standard trainee workday for maximal exposure to leadership development content and community building which is difficult to achieve in a fully virtual format.

Further directions include scaling up this model to a multi-institutional longitudinal presence as well as prospectively tracking the cohort of participants to see if WILD alumnae continue to stay in academic medicine and/or pursue leadership positions. Lastly, planning for such programs must include institutional financial and curricular support to increase recurring participation to achieve the stated objectives of the program and achieve longitudinal and durable success.

## Conclusion

In conclusion, WILD is an innovative women’s leadership model focusing specifically on trainees in an attempt to stave off the ‘leaky pipeline’ that has successfully pivoted to a hybrid format during the pandemic and sustained for 5 years.

**Table 1:** WILD curriculum overview

Session Title & Format	Objectives
**Empathy** Large-group lecture	1. Discuss the role of empathy as it relates to women physician leaders 2. Develop an understanding of how resilience relates to women physician leaders
**Using Family Leave & Interviewing While Pregnant** Large-group lecture	1. Formulate a plan of action items for taking family/parental leave 2. Identify trainees’ rights as it relates to family/parental leave 3. Review data on the “leaky pipeline” of women in academic careers
**Getting to YES! Negotiating Your Contract** Large-group lecture & small group workshop	1. Describe disparities that result from gender differences in negotiation practices 2. Identify the negotiable elements of a future contract to determine what to ask for 3. Name strategies that improve success in career negotiations
**Networking and Mentorship** Large-group lecture & small group workshop	1. Describe strategies to leverage professional networks to garner political influence 2. Describe how she can be an effective mentee to get the most out of a mentorship relationship
**Public Speaking Skills** Large-group lecture & small group workshop	1. Identify common public speaking pitfalls and how gender influences them 2. Practice specific public speaking elements through pair exercises
**Intersectionality in Medicine** Large-group lecture & small group workshop	1. Consider the intersection between gender and other identity categories in developing a leadership identity 2. Identify specific examples of how gender identity and other identity categories impact her personal leadership identity
**Responding to Microaggressions in Medicine** Large-group lecture & small group workshop	1. Develop an awareness of how gender bias manifests in her field of practice 2. Discuss strategies to navigate difficult workplace conversations regarding gender bias and/or microaggressions
**Building your Personal Brand** Large-group lecture & small group workshop	1. Draft a short personal mission statement 2. Practice an “elevator pitch” for trainees to sell themselves to a partner
**Allyship in Academic Medicine** Large-group lecture & small group workshop	1. Identify situations in which she may effectively employ allies during medical training 2. Compare and contrast the roles of marginalized individuals and allies in confronting bias in academic medicine
**CV Workshop** Large-group lecture & pair-share activity	1. Describe common pitfalls women make on their CVs 2. Identify actionable ways to “sell herself” more strongly on her CV
**Building Financial Power** Panel discussion	1. Name actionable strategies to increase her financial power 2. Identify institutional and outside resources to obtain financial information 3. Develop community with physician mentors through panel discussion
**How the Pandemic is Impacting Women: COVID-19** Large-group lecture & small group workshop	1. Discuss as a group the ways that COVID-19 has impacted career advancement, research productivity, clinical learning, and family planning 2. Grasp the outsized impact that the COVID-19 pandemic has had on women 3. Build community during a personally and professionally challenging time
**Voting as Medicine** Large-group lecture	1. Describe ways in which trainees can incorporate promotion of civic engagement into their clinical practice 2. Describe educational opportunities for physicians to advocate for their patients on a policy level
**Building your Board of Directors** Large-group lecture & small group workshop	1. Describe strategies to leverage professional networks to garner political influence 2. Describe how she can be an effective mentee to get the most out of a mentorship relationship
**Fertility in Medicine** Large-group lecture & panel discussion	1. Describe fertility challenges experienced as a result of medical training 2. Discuss the fertility options available at UCSF

**Table 2:** Participant confidence in leadership competencies and short-term effectiveness of WILD sessions

**“I feel confident in…”**	**Corresponding WILD session**	Mean Confidence Expressed on a 5-point Likert Scale	
		**Baseline**	**Post-session**
Using my mentor or coach to help my leadership effectiveness	Networking and Mentorship	3.38	4.43 (*p* < 0.001)
Dealing with micro-aggressions	Responding to Microaggressions in Medicine	3.04	4.43 (*p* < 0.001)
Developing my personal mission statement	Building your Personal Brand	3.28	4.38 (*p* < 0.001)
Public speaking skills	Public Speaking Skills	3.54	4.11 (*p* < 0.001)
Understanding my rights in terms of family leave	Using Family Leave & Interviewing While Pregnant	2.52	3.79 (*p* < 0.001)
Negotiation skills for a job, a promotion, a raise, space, leadership positions, etc.	Getting to YES!: Negotiating your Contract	2.29	3.75 (*p* < 0.001)
Garnering political influence	Building your Board of Directors	2.48	3.71 (*p* < 0.001)

### Electronic supplementary material

Below is the link to the electronic supplementary material.

Supplementary Material 1

Supplementary Material 2

## Data Availability

The datasets used and/or analyzed during the current study available from the corresponding author on reasonable request.
