# Supplementary material for: Creation of a sustainable longitudinal women in Leadership Development (WILD) curriculum focused on graduate medical education trainees
Source: BMC Med Educ. 2024 Apr 5;24:374. doi: 10.1186/s12909-024-05369-3 (PMC10996076; doi:10.1186/s12909-024-05369-3)
Supplement: Supplementary file 1 — Supplementary Material 1 [file 12909_2024_5369_MOESM1_ESM.pdf]

Supplemental Figure Citations

1. *What Makes Life Worth Living in the Face of Death.*; 1494946863. Accessed June 13, 2022. https://www.ted.com/talks/lucy_kalanithi_what_makes_life_worth_living_in_the_face_of_death

2. Collins C. *Making Motherhood Work: How Women Manage Careers and Caregiving*. Princeton University Press; 2019. https://doi.org/10.2307/j.ctvc7730x

3. Collins C. Two New Moms Return to Work — One in Seattle, One in Stockholm. *Harvard Business Review*. Published online March 3, 2020. Accessed June 13, 2022. https://hbr.org/2020/03/two-new-moms-return-to-work-one-in-seattle-one-in-stockholm

4. Berman RA, Gottlieb AS. Job Negotiations in Academic Medicine: Building a Competency-Based Roadmap for Residents and Fellows. *J Gen Intern Med*. 2019;34(1):146-149. doi:10.1007/s11606-018-4632-2

5. Fisher R, William Ury, Patton, William. *Getting to Yes: Negotiation Agreement Without Giving In*. Penguin Books; 2011.

6. Shen MR, Tzioumis E, Andersen E, et al. Impact of Mentoring on Academic Career Success for Women in Medicine: A Systematic Review. *Acad Med*. 2022;97(3):444-458. doi:10.1097/ACM.0000000000004563

7. Farkas AH, Bonifacino E, Turner R, Tilstra SA, Corbelli JA. Mentorship of Women in Academic Medicine: a Systematic Review. *J Gen Intern Med*. 2019;34(7):1322-1329. doi:10.1007/s11606-019-04955-2

8. Alter CH. *The Credibility Code: How to Project Confidence and Competence When It Matters Most*. Meritus Books; 2012.

9. Mahoney MR, Wilson E, Odom KL, Flowers L, Adler SR. Minority Faculty Voices on Diversity in Academic Medicine: Perspectives From One School. *Acad Med*. 2008;83(8):781-786. doi:10.1097/ACM.0b013e31817ec002

10. Torres MB, Salles A, Cochran A. Recognizing and Reacting to Microaggressions in Medicine and Surgery. *JAMA Surgery*. 2019;154(9):868-872. doi:10.1001/jamasurg.2019.1648

11. Exley C, Kessler J. Why Don’t Women Self-Promote As Much As Men? *Harvard Business Review*. Published online December 19, 2019. Accessed June 13, 2022. https://hbr.org/2019/12/why-dont-women-self-promote-as-much-as-men

12. ReadySet Ally Skills Workshop. Diversity, Equity & Inclusion Consulting — ReadySet. Accessed June 13, 2022. https://www.thereadyset.co/ally-skills-workshop

13. COVID-Personal-Statement-and-CV-Guidance.pdf. Accessed June 13, 2022. https://facultyacademicaffairs.ucsf.edu/academic-personnel/academic-review-and-advancement/COVID-Personal-Statement-and-CV-Guidance.pdf

14. Creating a standout CV | Medical CV examples and tips | AMA. Accessed June 13, 2022. https://www.ama-assn.org/residents-students/career-planning-resource/creating-standout-cv

15. Parent A. Creating a Physician CV That Shines. NEJM CareerCenter Resources. Published August 8, 2018. Accessed June 13, 2022. https://resources.nejmcareercenter.org/article/creating-a-physician-cv-that-shines/

16. Molldrem C, McDuffee PS. *The Physician's Guide to Financial Independence*. self-published; 2018.

17. Alon T, Doepke M, Olmstead-Rumsey J, Tertilt M. *The Impact of COVID-19 on Gender Equality*. National Bureau of Economic Research; 2020. doi:10.3386/w26947

18. #225 Women in Medicine: COVID Edition. The Curbsiders. Published July 16, 2020. Accessed June 13, 2022. https://thecurbsiders.com/podcast/225

19. About. The VOICE Project. Accessed June 13, 2022. https://voiceproject.ucsf.edu/about

20. Mentors and sponsors and peers, oh my! Sarkar Lab. Accessed June 13, 2022. https://sarkarlab.ucsf.edu/new-blog/2018/12/3/mentors-and-sponsors-and-peers-oh-my

21. Mroz J. A Medical Career, at a Cost: Infertility. *The New York Times*. https://www.nytimes.com/2021/09/13/health/women-doctors-infertility.html. Published September 13, 2021. Accessed June 28, 2022.

22. Marshall AL, Arora VM, Salles A. Physician Fertility: A Call to Action. *Acad Med*. 2020;95(5):679-681. doi:10.1097/ACM.0000000000003079
