# Supplementary material for: Creation of a sustainable longitudinal women in Leadership Development (WILD) curriculum focused on graduate medical education trainees
Source: BMC Med Educ. 2024 Apr 5;24:374. doi: 10.1186/s12909-024-05369-3 (PMC10996076; doi:10.1186/s12909-024-05369-3)
Supplement: Supplementary file 2 — Supplementary Material 2 [file 12909_2024_5369_MOESM2_ESM.pdf]

Supplemental Table 1: Key resources in WILD session content development

Session Title	Key Resources
**Empathy**	"What Makes Life Worth Living in the Face of Death" 2016 TED talk by Lucy Kalanithi[1]
**Effectively Using Family Leave & Interviewing While Pregnant**	"Making Motherhood Work: How Women Manage Careers and Caregiving" 2019 book by Caitlyn Collins[2]<br><br>"Two New Moms Return to Work – One in Seattle, One in Stockholm" 2020 Harvard Business Review article by Caitlyn Collins[3]
**Getting to YES!: Negotiating Your Contract**	"Job Negotiations in Academic Medicine: Building a Competency-Based Roadmap for Residents and Fellows"[4]<br><br>"Getting to Yes: Negotiation Agreement Without Giving In" book by Roger Fisher and William Ury[5]
**Networking and mentorship**	"Impact of Mentoring on Academic Career Success for Women in Medicine: A Systematic Review" Academic Medicine article by Mary Shen, et. al.[6]<br><br>Mentorship of Women in Academic Medicine: A Systematic Review" Journal of General Internal Medicine article by Amy Farkas, et. al.[7]
**Public Speaking Skills**	"The Credibility Code: How to Project Confidence and Competence When it Matters Most" - book by Cara Alter[8]
**Intersectionality in Academic Medicine**	"Minority Faculty Voices on Diversity in Academic Medicine: Perspectives From One School" – Megan Mahoney[9]
**Seeing and Responding to Microaggressions in Academic Medicine**	"Recognizing and Reacting to Microaggressions in Medicine and Surgery" – JAMA Surgery article by Madeline Torres, et. al.[10]
**Building your Personal Brand**	"Elderhood: Redefining Aging, Transforming Medicine, Reimagining Life" book by Lousie Aronson<br><br>"Why don't women self-promote as much as men?" – Harvard Business Review article by Christine Exley and Judd Kessler[11]
**Allyship in Academic Medicine**	ReadySet DEI Consulting Firm Ally Skills Workshop[12]
**CV Workshop**	UCSF Faculty guidelines on how to address the impact of COVID-19 on your CV[13]<br>AMA "Creating a standout CV"[14]<br>NEJM "Creating a Physician CV that Shines"[15]
**Financial Power for Women in Medicine**	"Physician's Guide to Financial Independence" – book by Shaun McDuffee and Craig Molldrem[16]
**How the Pandemic is Impacting Women: COVID-19**	"The Impact of COVID-19 on Gender Equality" – National Bureau of Economic Research[17]<br><br>"#225 Women in Medicine: COVID Edition" The Curbsiders Podcast[18]
**Voting as Medicine**	The Voice Project at UCSF[19]
**Building your Board of Directors**	"Mentors and sponsors and peers, oh my!"- professional blog by Urmimala Sarkar[20]
**Fertility in Medicine**	"A Medical Career, at a Cost: Infertility", New York Times article by Jacqueline Mroz[21]<br><br>"Physician Fertility, A Call to Action", Academic Medicine Article by Ariela Marshall, et. al[22]

Supplemental Table 2: WILD focus group interview guide

<u>QUESTIONS</u>

Opening Questions:

1. I would like to start by asking you What motivated you to get involved with WILD?

Introduction Questions:

2. How do you define leadership and how do you see leadership playing a role in your career?

Key Questions:

3. Did your experience with WILD influence your leadership ability, and if so, how?
4. Did your experience in WILD affect the way you see yourself as a leader?
5. How, if at all, did your experience with WILD affect your connection to other women trainees at UCSF?
6. How, if at all, did WILD impact your wellbeing?
7. Was the WILD environment respectful of unique aspects of your identity (i.e. Race, gender identity, sexual orientation, etc.)
    - *Probe:* How could WILD be more supportive and/or inclusive with regard to these parts of your identity?

Transition Questions:

8. Please tell me about your overall experience with WILD this year, both positive and negative.
    - *Probe:* What was your favorite part of WILD, or the part most useful to you?
    - *Probe:* What was your least favorite part of WILD, or the part least useful to you?

Closing Questions:

9. What made it easy or hard for you to attend sessions in the program?
10. What sessions do you wish WILD had put on this year and why?
    - *Prompt: The sessions were: empathy, family leave rights and resources, negotiation skills, networking and mentorship, public speaking, microaggressions, building your brand).*
11. Do you have any questions for me, or additional comments that you'd like to make?

Supplemental Table 3: WILD participant characteristics

Characteristic	Number of participants n (% of 291)
**Specialty Department**	
Internal Medicine and subspecialties	153 (53%)
Pediatrics and subspecialties	28 (10%)
Neurology and subspecialties	23 (8%)
Psychiatry and subspecialties	16 (5%)
Pathology and subspecialties	15 (5%)
General Surgery and subspecialties	15 (5%)
Dermatology and subspecialties	9 (3%)
Other	32 (11%)
**Stage of Training**	
Fellow	124 (43%)
Resident	112 (39%)
Chief Resident	35 (12%)
Other	19 (7%)
**Race/Ethnicity**	
Non-Hispanic White	146 (50%)
Asian/Pacific Islander	82 (30%)
Black/African American	29 (10%)
Hispanic/Latinx	13 (4%)
Other	12 (4%)
Native American	3 (1%)
Prefer not to state	2 (1%)

Supplemental Table 4: Number of sessions attended per WILD participant

Number of sessions attended per participant	Number of participants (n, % of 291)
1	231, 79.3%
2	35, 12.0%
3	13, 4.4%
4	8, 2.7%
5	2, 0.7%
6	2, 0.7%

Supplemental Table 5: Attendance at WILD sessions

Session Name	# of participants attended
Empathy	47
Using Family Leave & Interviewing While Pregnant	22
Getting to YES!: Negotiating Your Contract #1	43
Networking and mentorship	18
Public Speaking Skills	79
Intersectionality in Medicine	16
Responding to Microaggressions in Medicine	23
Building your Personal Brand	16
Allyship in Academic Medicine #1	13
Getting to YES! Negotiating Your Contract #2	47
CV Workshop	26
Building Financial Power	23
How the Pandemic is Impacting Women: COVID-19	11
Voting as Medicine	6
Building your Board of Directors	14
Allyship in Academic Medicine #2	11
Fertility in Medicine	14

Supplemental Table 6: Representative quotes and themes of WILD focus groups

Theme/Subtheme	Representative quotes
**Community**	
Addresses diversity of experiences	A lot of women in medicine or women in surgery or women in whatever always seem to gravitate towards motherhood. I totally agree, and so I don't feel that that happened in WILD, which was appreciated because I don't have children and it's not something that right now is a really big concern of mine, so it would be not so relevant if I was heading to these sessions after long days to then have those discussions. Although very important for women that do have children because that's a really challenging item to balance, I think that not having that as the sole focus was refreshing.
Institutional structured support	And then also hinge those informal conversations and informal experiences on a more formal, curricular background has been really helpful.
Platform to process experiences in medical training	So, from just being able to review frustrating things that happened in the day with someone who was maybe there or kind of understands. And then also when you're starting to feel really frustrated at the dysfunction of your own department, to know that everybody is experiencing a variation on the same exact theme as you, I think really helps foster that sense of community. So, I found it really helpful from that standpoint.
Safe space	Yeah, I think there's advantages to both, like having a trainee oriented environment, where there isn't faculty and attendings. And I think that's just because... it just kind of changes... a lot of them are your bosses and it just changes the dynamic in the room. I feel like whenever I'm talking to someone in more of like a leadership position, I'm just... Not omitting things, but just being more mindful and careful of, what I'm saying, versus just having it be a more relaxed environment.
Attuned to trainee needs	I feel like the topics are just so relevant, and so I think relevancy of the themes that are discussed is huge. Not only as a trainee, but particularly as a female woman trainee, I think you guys are really hitting the highlights of things that anyone would want to know
**Empowerment**	
Expectations in medical training	I think one deficit is we're never really taught how to do that, you know, how to lead. I think leadership can mean so many things. It can mean leading your clinical team, it can be leading your research team. You could actually have some sort of administrative role in a program, like I know how you have your program director of fellowship and everything. So, I think so many different things can be led, and yet, I don't know about y'all, but I don't think I've ever taken a leadership course ever.

Fills a gap in training	I was thinking about how do we learn leadership, right? It's like there's intentional explicit training, which some people have done and some people haven't done, but it's not like we all go to an MBA or a business school to learn how to be a leader. We don't do that.
Identity formation	I think that that's what I saw these sessions tried to focus on, the non-clinical items that are really important for us to be able to thrive and to feel like we are able to do our jobs to the fullest and still feel like a complete human being
Prior interest in leadership	For me, I'll say I have always had that personal tendency to take on some type of leadership role and definitely something that I will continue to strive for in positions ahead.
Work-life balance	We are all thinking about how to get jobs. Many of us are thinking about how to plan for children. There's common themes that just as women in medicine, I think we all experience, and so that was really nice across specialty divides to have that.
**Leadership Skills**	
Redefining leadership	I feel like before WILD, they definitely saw like "self-promotion" as a real, unattractive thing. Like an unattractive quality that I tried to not apply to myself. And I feel like through WiLD, not that it's about like, "Oh I'm so great" or that sort of thing, but it's definitely about advocating for who you are, what you've achieved and letting people know but not in a very brag - Like I felt like it helped me gain strategies in how to express, what I'm doing so that people in other leadership roles can take notice. And I'm seeing role models talk about how they engaged in promoting themselves, I felt it's really helpful.
Self-awareness	They were very attuned and aware of all of the different ways that you can blunder discussions about race, religion, gender, sexual orientation, and they were very ... To the extent where I was like, "Gosh, do I regularly do x, y, or z? Do I always say what my preferred pronouns are? What's the harm in not saying what your preferred pronouns are?"
Skill building	Yeah, I would say it's been probably like pretty subtle, small movements. I would say when I've been in conversations with a chair of a department or a program director or something like that. Someone who's kind of high up, I feel like I've been more empowered to just say, Hey, I'm working on this project and this project, and have more of an elevator pitch-type-thing, just so people know what I've been working on.
**Mentorship & Networking**	
Benefits of near peer mentorship	It resonates with me also because we share experiences that may not necessarily resonate with people in other stages in their life. We share a lot of dynamics of power and also figuring out what you're doing and what your next steps are and learning what your role is.

Networking	(...) identified women leaders within our community for which, I know are advocates for us and, are also in positions of power. And so people that you can sometimes go to if there's like a specific instance or something that you feel you would appreciate more support on, or something like that. It basically just uncovered this whole infrastructure that I never knew existed until I joined the group.
Role model	(...) one thing that probably struck me was just the speakers themselves, as being examples, like seeming relatable, seeming that you could see yourself in the roles or accomplishments that they had attained, and so therefore it felt attainable to you as well to be successful and to hold these various roles, balancing a variety of things in their lives.
